# The first genome sequence of *Anopheles squamous* from Macha, Zambia

**DOI:** 10.12688/f1000research.130734.1

**Published:** 2023-03-24

**Authors:** Valerie T. Nguyen, Travis C. Collier, Sangwoo Seok, Xiaodi Wang, Monicah M. Mburu, Limonty Simubali, Mary E. Gebhardt, Douglas E. Norris, Yoosook Lee

**Affiliations:** 1 Florida Medical Entomology Laboratory, Department of Entomology and Nematology, Institute of Food and Agricultural Sciences, University of Florida, Gainesville, Florida, 32962, USA; 2 Independent researcher, Vero Beach, FL, 32962, USA; 3 Macha Research Trust, Macha, Southern Province, Zambia; 4 The W. Harry Feinstone Department of Molecular Microbiology and Immunology, The Johns Hopkins Malaria Research Institute, Johns Hopkins Bloomberg School of Public Health, Johns Hopkins University, Baltimore, Maryland, 21205, USA

**Keywords:** Anopheles squamosus, understudied malaria vector, Africa, Zambia

## Abstract

Despite efforts to minimize the impacts of malaria and reduce the number of primary vectors, malaria has yet to be eliminated in Zambia. Understudied vector species may perpetuate malaria transmission in pre-elimination settings. *Anopheles squamosus* is one of the most abundantly caught mosquito species in southern Zambia and has previously been found with *Plasmodium falciparum* sporozoites, a causal agent of human malaria. This species may be a critical vector of malaria transmission, however, there is a lack of genetic information available for *An. squamosus.* We report the first genome data and the first complete mitogenome (Mt) sequence of *An. squamosus.* The sequence was extracted from one individual mosquito from the Chidakwa area in Macha, Zambia. The raw reads were obtained using Illumina Novaseq 6000 and assembled through NOVOplasty alignment with related species. The length of the *An. squamosus* Mt was 15,351 bp, with 77.9 % AT content. The closest match to the whole mitochondrial genome in the phylogenetic tree is the African malaria mosquito, *Anopheles gambiae.* Its genome data is available through National Center for Biotechnology Information (NCBI) Sequencing Reads Archive (SRA) with accession number SRR22114392. The mitochondrial genome was deposited in NCBI GenBank with the accession number OP776919. The ITS2 containing contig sequence was deposited in GenBank with the accession number OQ241725. Mitogenome annotation and a phylogenetic tree with related *Anopheles* mosquito species are provided.

## Introduction

**Figure 1.**
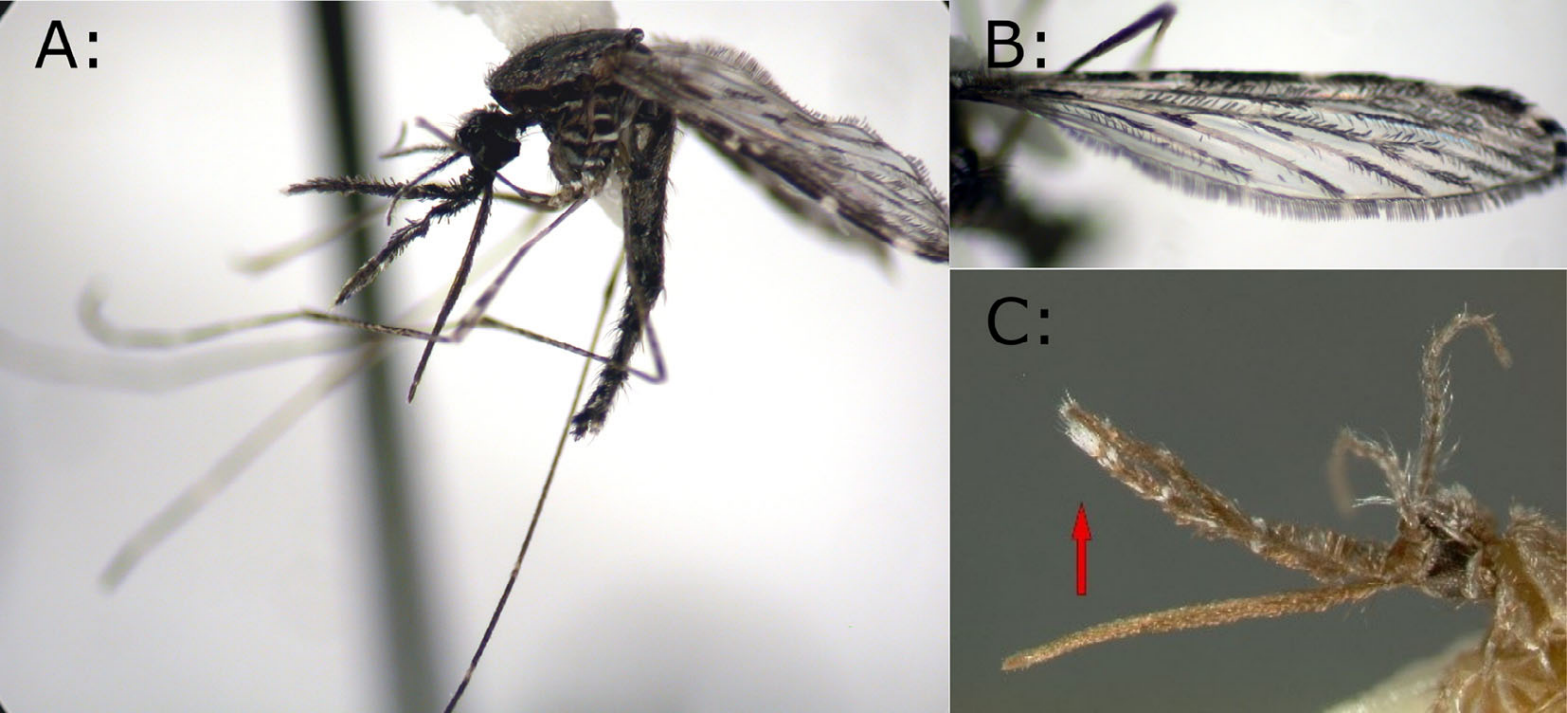
A: *Anopheles squamosus* image. B: *An. squamosus* wing. C: Head image of *An. squamosus*, one of the features used for species identification. A and B have been reproduced with permission from Dr. Rebekah Kading (Colorado State University). ^21^ C has been reproduced with permission from Walter Reed Biosystematics Unit (WRBU). ^22^

*Anopheles squamosus* (Theobald, 1901; Figure 1) can be found across Africa ^1^ and is of particular relevance to public health due to its implication in the spread of residual malaria cases. *Anopheles squamosus* is one of the most abundantly caught anopheline species in malaria vector surveillance studies in southern Zambia. However, it is understudied species because of its exophilic and zoophilic behaviours. ^2^^,^^3^ Though they are predominantly associated as a zoophilic species, they have been discovered to have high anthropophily in southern Zambia. ^4^ Additionally, there has been the detection of *Plasmodium falciparum* sporozoite and DNA, a causal agent of human malaria, in *An. squamosus.* ^2^^,^^5^

Unfortunately, there are two key barriers to pursuing the rigorous investigation of the epidemiologically important traits of this vector, such as host choice, biting behaviours, and dispersal capacity. First, *An. squamosus* is morphologically indistinguishable from *An. cydippis* at the adult stage. Although they are morphologically distinct as larvae, larvae are often difficult to locate in abundance. There are numerous examples of sympatric *Anopheles* sibling species expressing drastic differences in insecticide resistance ^6^ or host choice. ^7^^,^^8^ These differences make species confirmation critical to assessing and mitigating malaria transmission risk. Second, there is limited genetic information (173 sequences total in GenBank as of August 2022) for *An. squamosus*, most (N=166; 96%), are partial sequences of the mitochondrial cytochrome c oxidase subunit I (COI) gene. ITS2 sequences are better at differentiating species within a complex than COI sequences ^9^ but existing ITS2 primers do not typically work on *An. squamosus* and the absence of sequence data for this region prohibit the design of functional diagnostic PCR primers.

To overcome these two barriers and advance investigative efforts aimed at this widespread, yet neglected malaria vector, we carried out the first Illumina high-throughput sequencing of this species.

## Methods

### Data collection

The *An. squamosus* sample used for the genome sequencing was collected in Chidakwa near Macha, Zambia (utm-x: 0478202, utm-y: 8184394) using a CDC light trap placed outdoors near a goat pen. Samples were frozen after collection at –20°C until DNA extraction. DNA was extracted using a magnetic bead-based protocol optimized for mosquito DNA for Next-generation sequencing. ^10^ The head and thorax were dissected from the sample and hydrated in nuclease-free water for 1 hour at 4°C. Tissues were then removed from the water and homogenised in a mixture of 2 μL Proteinase K (100 mg/mL) and 98 μL PK Buffer in a 1.5 mL Eppendorf microcentrifuge tube (add company name), followed by incubation at 56°C for 2 hours. The lysate was transferred to a new 1.5 mL microcentrifuge tube and mixed with a MagAttract Mix consisting of 100 μL isopropanol, 100 μL Buffer AL, and 15 μL MagAttract Suspension G (Qiagen, Hilden, Germany). The mixture was incubated at room temperature for 10 minutes and occasionally vortexed to ensure that the magnetic beads were evenly dispersed. The microcentrifuge tube containing the lysate was then moved to a magnetic bead separator until the liquid appeared clear. After a series of ethanol washes of magnetic beads, DNA was eluted from the beads with 100 μL AE Buffer and stored at −20°C until library preparation. The library preparation was completed using the QIAseq FX UDI kit (Qiagen, Hilden, Germany) using 20 ng genomic DNA as input for the protocol as previously described. ^11^

### Data analysis

**Figure 2.**
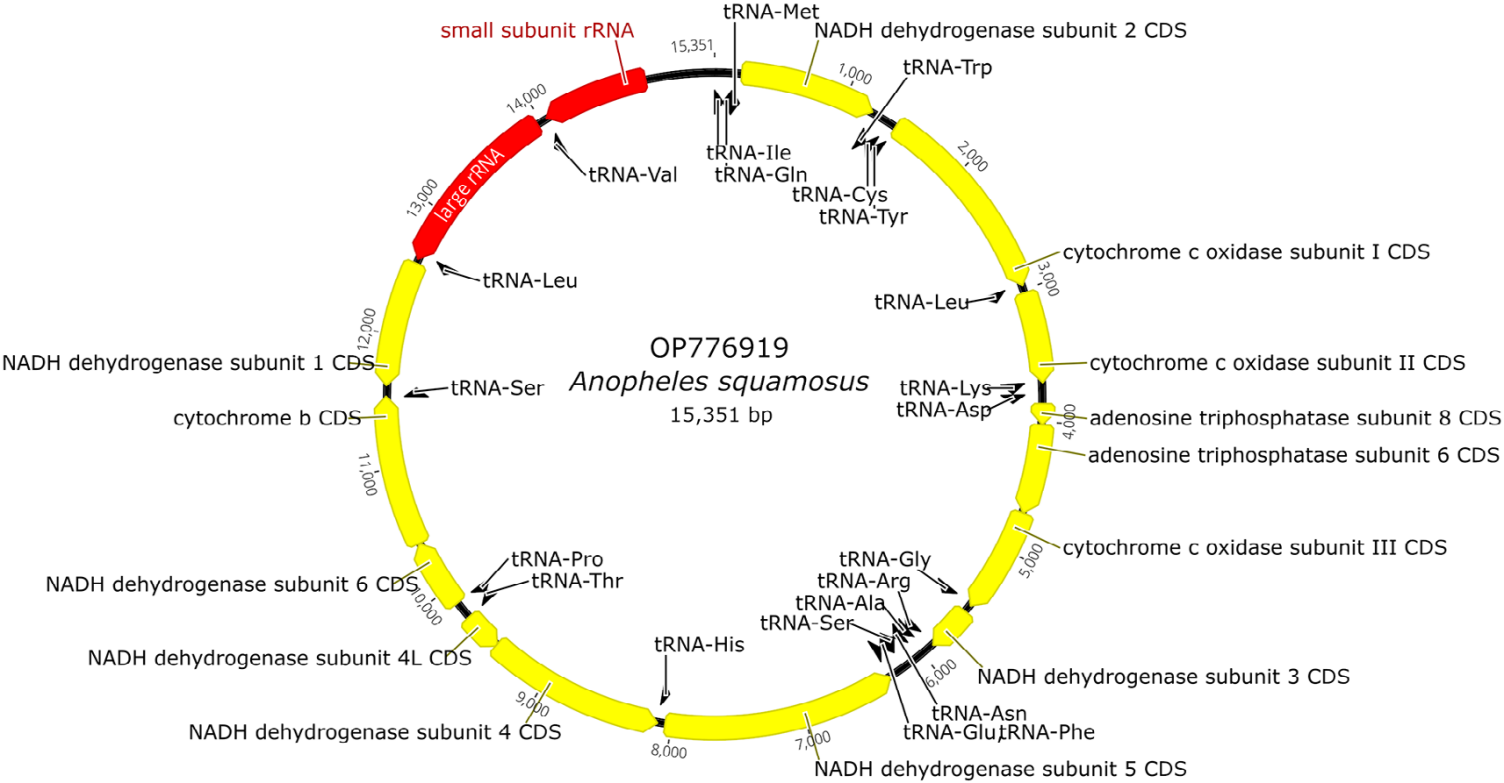
Mitogenome map of *An. squamosus* including annotated genes.

Raw sequencing reads were trimmed using fastp (RRID:SCR_016962) version 0.20.1. ^12^ Mitogenome (Mt) contig was assembled using NOVOPlasty (RRID:SCR_017335) version 4.3.1. ^13^ Automatic annotation of mitogenome was conducted with the MITOS website ^14^ using the invertebrate genetic code for mitochondria under default settings. Some automatic annotations were not consistent with typical *Anopheles* mitochondrial gene start and/or end positions. Manual adjustments were made to inconsistent automatic annotations by shifting the start and end positions to match existing *Anopheles* mitochondrial gene annotations found in GenBank. Annotation information was also deposited to the GenBank with the genome sequence. The full genomic map is provided in Figure 2.

Phylogenetic analysis was conducted using the mitogenome sequences of seven *Anopheles* species and one *Aedes* species as an outgroup in **.** The Jukes-Cantor model was used to calculate the pairwise genetic distances and the neighbour-joining method was used to build the phylogenetic tree in Geneious Prime (RRID:SCR_010519) 2022.02 (Biomatters, Auckland, New Zealand) ^15^ (free alternative, AliView).

Draft genome assembly was conducted using MaSuRCA (RRID:SCR_010691) version 4.0.9 ^16^ in order to find a contig containing Internal transcribed spacer 2 (ITS2) sequence. Basic local alignment search tool (BLAST) (RRID:SCR_004870) was used for the resulting contigs to locate contigs with highest similarity with only *An. squamosus* ITS2 sequence available on GenBank (accession number MK592071).

## Results

We yielded 105 million reads from sequencing a single *An. squamosus* sample. Of these, 238,740 reads were used to assemble mitochondrial genome. Draft genome assembly using MaSuRCA produced 58,252 scaffolds with the total size of scaffolds of 350Mbp. N50 scaffold length was 21,439bp. Among these contigs, we identified one contig containing ITS2 region (GenBank accession number OQ241725), which was 1,223 bp long.

The length of the *An. squamosus* Mt (GenBank accession number OP776919 ^23^) was 15,351 bp and the percentage A+T was 77.9% ( Figure 2). The average A+T percentage of eight other anopheline species was 77.7% (±0.61 SD). The length of this mitochondrial genome was a similar length to other anopheline species that have been deposited in GenBank, with the average of the eight species compared in this analysis being 15,363 bp. The content for this mitochondrial genome includes two ribosomal RNAs, 22 transfer RNAs, and 35 protein-coding genes. The cytochrome c oxidase I (COI) fragment spanning 1,462-2,132 bp of *An. squamosus* sequence had 97.7% (±4.27 SD, N=9) similarity to the COI sequence of *An. squamosus* deposited in GenBank.

**Figure 3.**
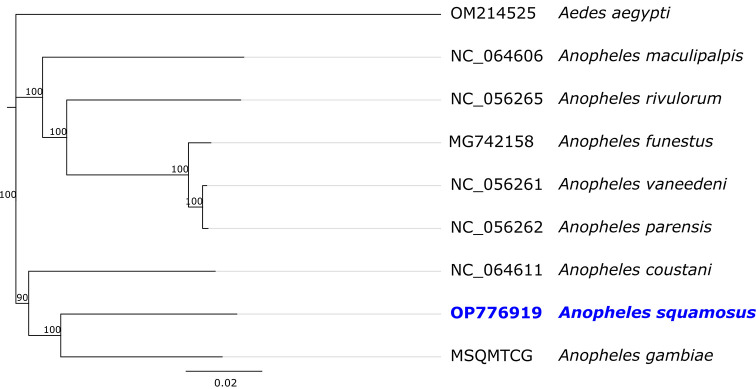
Phylogenetic tree based on mitogenome sequences of *An. squamosus* and its related mosquitoes. Species names are provided next to the GenBank accession numbers. Numbers at nodes indicate bootstrap values out of 100 replicates. *Aedes aegypti* was considered as an outgroup. The scale bar indicates relative nucleotide difference (0.02=2% nucleotide difference).

In the phylogenetic analysis ( Figure 3), the closest match to the whole mitogenome sequence of *An. squamosus* was the African malaria mosquito *An. gambiae* (GenBank accession number L20934), with 91.5% sequence similarity. This comparable sample was identified as *An. gambiae* and published in 1993 before *An. gambiae* were separated into two species: *An. gambiae* and *An. coluzzii.* ^17^ Nevertheless, previous studies suggest that mitogenome sequence alone is not sufficient to distinguish *An. gambiae* s.s. from *An. coluzzii.* ^18^^,^^19^

This study provides a critical genomic resource for research of this understudied malaria vector. Our short reads sequencing data was not sufficient to assemble high-quality reference genome and revealed the need for alternative long-read sequencing technology for a high-quality genome assembly. However, we provided a key ITS2 region data that researchers can develop a low-cost molecular diagnostic assay to identify species. Currently available ITS2 primers for anopheline species identification typically does not produce a PCR amplicon, which is one of the major roadblocks in carrying out surveillance and research of this species. We identified the ITS2-containing contig (GenBank Accession number OQ241725) that could be used for new primer design that would amply the ITS2 fragments more reliably for *An. squamosus.* Our genome sequence data could be used for further variant identification once high-quality reference genome become available for *An. squamosus.* The mitogenome sequence could also be used to identify phylogenetic relationship within and between related species and infer gene flow/dispersal. ^9^^,^^20^

### Ethical considerations

The study involves collection of mosquito specimen near goat pens within individual households in Chidakwa, Zambia as part of the project that had been approved by National Health Research Authority, Zambia: Approval No: NHRA00016/18/08/2021.

## Data Availability

GenBank: *Anopheles squamosus* mitochondrion, complete genome. Accession number OP776919; https://identifiers.org/ncbi/insdc:OP776919. ^23^ BioProject: Complete mitogenome sequence of *Anopheles squamosus* from Macha, Zambia. Accession number PRJNA896235; https://identifiers.org/bioproject:PRJNA896235. ^24^ SRA: DNA-Seq of mosquito *Anopheles squamosus.* Accession number SRR22114392; https://identifiers.org/insdc.sra:SRR22114392. ^25^ BioSample: *Anopheles squamosus* isolate As22MACHA01. Accession number SAMN31538381; https://identifiers.org/biosample:SAMN31538381. ^26^
