# Supplementary material for: The first genome sequence of Anopheles squamous from Macha, Zambia
Source: F1000Res. 2023 Mar 24;12:330. [Version 1] doi: 10.12688/f1000research.130734.1 (PMC10568211; doi:10.12688/f1000research.130734.1)
Supplement: Peer review history

# Reviewer report: Reviewer response for version 1

**DOI:** 10.5256/f1000research.143510.r200578

**Published:** 2023-10-11

**Authors:** Tara Roth

**Affiliations:** 1 San Mateo County Mosquito and Vector Control District, Burlingame, California, USA

This article details the production of the An. squamosus full genome which is intended to provide a useful reference for future researchers to investigate the role this species may play in the ecologic cycle of malaria in Zambia. The article stresses that mitogenome sequences (predominantly CO1) are not sufficient to differentiate closely related species which is a consistent issue across Culicidae. They also stress that primers for the alternative region, ITS2, do not amplify An. squamosus. The article is well written, however there are a few typos that should be addressed. Under Methods -> Data Collection, the line "Eppendorf microcentrifuge tube (add company name)" should be fixed. The final paragraph of the results section could be tightened up a bit - there are a few minor grammatical errors (eg: "The mitogenome sequence could also be used to identify phylogenetic relationship within...")

Are the datasets clearly presented in a usable and accessible format, and the assembly and annotation available in an appropriate subject-specific repository?

Yes

Are sufficient details of the sequencing and extraction, software used, and materials provided to allow replication by others?

Yes

Are the rationale for sequencing the genome and the species significance clearly described?

Yes

Are the protocols appropriate and is the work technically sound?

Yes

Reviewer Expertise:

I am a vector ecologist with a specialization in mosquitoes and ticks. I am currently working on a project addressing the distribution of sister Anopheline species An. hermsi and An. freeborni in California and thus am familiar with the regions (CO1 and ITS2) being discussed in this paper

I confirm that I have read this submission and believe that I have an appropriate level of expertise to confirm that it is of an acceptable scientific standard.

# Reviewer report: Reviewer response for version 1

**DOI:** 10.5256/f1000research.143510.r167805

**Published:** 2023-04-05

**Authors:** Megan A. Riddin

**Affiliations:** 1 UP Institute for Sustainable Malaria Control (UP ISMC), Faculty of Health Sciences, University of Pretoria, Pretoria, South Africa

This research presented the first genome data and complete mitogenome sequence of *Anopheles squamosus*. *Anopheles squamosus* is commonly caught in abundance in Zambia and has been recently implicated in malaria transmission through the detection of P. falciparum circumsporozoites and through PCR. This research utilised a single *An. squamosus* specimen from Chidakwa, Zambia, which was collected through a CDC light trap placed outside near a goat pen. DNA was extracted from the specimen following a magnetic bead-based protocol specific for mosquito DNA for next generation sequencing. The library was then prepared using the eluted DNA and a standardised preparation kit (QIAseq FX UDI Kit). The sequencing reads were trimmed and mitogenome assembled using NOVOPlasty, and automatic annotation completed through the MITOS website. Phylogenetic analyses were performed with an *Aedes *species outgroup in Geneious Prime. Final draft genome assembly was conducted using MaSuRCA. 

 The methods resulted in 105 million reads and one contig was identified as containing an ITS2 region. The mitochondrial genome was assembled and resulted in 15351 bp with 77% A+T percentage. Phylogenetic analyses identified the closest mitogenome sequence match to *An. gambiae* which was sequenced prior to separation of the complex into sibling species. 

 The research enabled identification of key ITS2 region data for development of low-cost molecular diagnostic assays, despite the short reads not being sufficient for high-quality reference genome assembly. The outputs further included the identification of the phylogenetic relationships to related species through assembled mitogenome sequence.

 This study presented vital data on an understudied species that has recently been implicated in malaria transmission in Zambia. Malaria has experienced recent upsurges in Zambia and although the primary vectors are thought to be *An. funestus*, *An. gambiae* and *An. arabiensis*, the detection of higher abundance of *An. squamosus* and *An. coustani*, as well as their unexpected anthopophillic behaviour has lent support to their role in transmission. The recent detection of both *P. falciparum circumsporozoite* in *An. squamosus* has highlighted the necessity to study this species. The use of full genome sequencing is highly beneficial not only for phylogenetic analysis but further for development of rapid molecular identification techniques as was exhibited in this study. This study was able to provide data that will assist towards classification and identification of the species to better understand ecology, epidemiology and therefore role of this species in malaria transmission in Zambia.

 The Introduction and rationale of the study is clearly explained and may benefit from some clarification on the chromosomal types or mention of possible clades that have been eluded to. However, as such work and suggestions are unpublished, it would be very interesting to see how the authors progress with this research towards understanding the phylogeny of the understudied species. 

 The Methods and Results are clear and concise and the diagrams are further well structured and complimentary to the text.

 Very few edits and/or additions are suggested and these are included below:

- Introduction, Line 3: "However, it is an understudied..."
- Introduction, Paragraph 2: It would be helpful to the reader to have understand if there is information on *An. cydippis*. Is this known as a vector and does it have anthropophilic or zoophilic behaviours? 
- Introduction, Paragraph 2: No mention is made of the unpublished claims of Green & Hunt that state there are 5 chromosomal forms of *An. squamosus* and also the publications that elude to multiple clades. This may be interesting to include to lend support to how understudied the species is.
- Data collection, Line 6: "...Eppendorf microcentrifuge tube (add company name)...." should be addressed.
- Results, paragraph 4: This paragraph requires some grammar editing such as "..that could be used for new primer design that would amply the ITS2 ...." should be corrected to "amplify".

Overall this is a very interesting and important article and is recommended for indexing.

Are the datasets clearly presented in a usable and accessible format, and the assembly and annotation available in an appropriate subject-specific repository?

Yes

Are sufficient details of the sequencing and extraction, software used, and materials provided to allow replication by others?

Yes

Are the rationale for sequencing the genome and the species significance clearly described?

Yes

Are the protocols appropriate and is the work technically sound?

Yes

Reviewer Expertise:

Medical entomology, vector control, malaria, vector-borne disease

I confirm that I have read this submission and believe that I have an appropriate level of expertise to confirm that it is of an acceptable scientific standard.
